# Daily light exposure habits of youth with migraine: A prospective pilot study

**DOI:** 10.21203/rs.3.rs-6682653/v1

**Published:** 2025-06-03

**Authors:** Carlyn Patterson Gentile, Ryan Shah, Blanca Marquez de Prado, Nichelle Raj, Christina Szperka, Andrew Hershey, Geoffrey Aguirre

**Affiliations:** Children's Hospital of Philadelphia; Thomas Jefferson University; Children's Hospital of Philadelphia; Children's Hospital of Philadelphia; Children's Hospital of Philadelphia; Cincinnati Children's Hospital Medical Center; University of Pennsylvania

**Keywords:** migraine, adolescents, photophobia, light exposure, light logging, circadian cycle

## Abstract

Photophobia is a common symptom in youth with migraine, but it is unknown if it leads to light avoidant behavior, and if such behaviors worsen disease burden. We conducted a feasibility study between November and March 2024 measuring light exposure using wearable light logger pendants in 20 youth with migraine (10–21 years old) while migraine symptoms were tracked with a text-based daily diary. On average, participants received recommended light exposure during only 14.5% +/− SD 7.0 of daylight hours but were consistently below the recommended maximum light levels 3 hours prior to bed (77.5% +/− 21.6 of the time), and at night (99.1% +/− 2.9 of the time). Daily light exposure patterns that were phase shifted 60 minutes later in youth with chronic (compared to non-chronic) migraine. Measuring daily light exposure is feasible in pediatric populations with photophobia and reveals intriguing trends that warrant further study.

## Introduction

Variation in visual diet (i.e. the intensity and timing of light exposure during daily life) has been associated with health outcomes. Exposure to brighter days and darker nights is known to confer reduced mortality risk,^1^ perhaps related to the effect that light has upon circadian biology.^2^ Insufficient daytime melanopic illuminance (short wavelength “blue light”) and evening melanopic illuminance from artificial light both have the potential to disrupt circadian entrainment.^3,4^ In support of this, multiple studies have associated nighttime screen use with poorer health-related quality of life and sleep disruption.^5-7^

Photophobia (i.e. light sensitivity) is a common symptom of multiple neurologic and ophthalmologic conditions including migraine,^8 9^ which may influence the visual diet through avoidance of high intensity visual environments.^10^ There has, however, been limited empirical study of how visual diet is altered and potentially influences symptoms in people with photophobia. Recently developed, wearable light loggers now provide the ability to address these questions using quantified measures of visual diet during daily life.^11-13^ These small, battery-powered devices record the absolute level of light falling upon a detector over a period of many days. These recordings provide the relative amount of light across wavelengths, supporting inferences regarding the types of light encountered (natural vs. artificial) and the biological effect upon different classes of retinal photoreceptors (melanopsin containing cells vs. cones).

We measured the visual diet for 20 youth with migraine using wearable light loggers that continuously tracked ambient light exposure over 7 days during a typical week. We hypothesized that continuous measurement of the visual diet in youth with migraine would be feasible and yield intriguing associations with disease burden in youth with migraine. Specifically, we predicted that a restricted and poorly timed visual diet (i.e., overall less and poorly timed light) would be associated with worse photophobia, chronic migraine, and worse headache-related disability.

## Materials and Methods

### Study Design

This single-center prospective observational pilot study was conducted within the pediatric headache program within the Children’s Hospital of Philadelphia (CHOP) neurology department between October 2024 and March 2025. The protocol received approval from the Institutional Review Board at CHOP.

#### Participants

Potentially eligible participants were identified by screening patients being seen in upcoming headache clinics via chart review. Participants were included if they were between the ages 10 to 21 years, ICHD-3 defined migraine with or without aura given by a headache specialist of any headache frequency and consented (≥ 18 years) or assented with parent consent (< 18 years) to participate in the study. Exclusion criteria were history of major neurological conditions besides migraine (e.g. history of epilepsy, stroke, multiple sclerosis), recent history of concussion (< 3 months), or were starting or weaning off a headache preventive medication (supplement or prescription) without being on a stable dose for at least 1 month prior to enrollment.

#### Data Collection:

Devices were shipped to the participants’ home and once received, a virtual visit was conducted to review the proper use of the wearable devices and text-diary before recording began. At this visit, participants completed baseline questionnaires through REDCap (Research Electronic Data Capture)^14,15^ hosted by the institution. The first full day of recording (12 am – 11:59 pm) was considered “Day 1,” and data collection ran for 7 full days. During these 7 days, diary questions were texted between 7pm and 11pm based on participant preference. During data collection, participants continuously wore the light logger while responding to headache diary prompts each evening. The light logger was removed while sleeping to avoid choking or the light logging device being obscured by bedsheets. Participants were instructed to keep the light logger on a bedside table, which is standard for light logger studies.^16^ At a scheduled clinic visit after the recording week, participants returned the wearable devices and filled out REDCap-based questionnaires. If participants had personal eyewear, they wore to filter light (e.g. blue light blocking, sunglasses), the light transmittance of this personal eyewear was measured.

Demographic, medical history, clinical characteristics, and treatment were gathered. Standardized and validated survey data included the CHOP Headache Questionnaire,^17^ including number of any headache days per month and number of bad headache days per month. Light sensitivity was measured using the Visual Light Sensitivity Questionnaire (VLSQ-8), which is an 8-question validated measurement of light sensitivity with possible scores ranging from 8–40.^18^ Fear of Pain Questionnaire for Children (FOPQ-C) is a validated metric which was used to measure fear-avoidant responses to pain. After the week of data collection, headache-related disability over the previous month was assessed with the Pediatric Migraine Disability Assessment (PedMIDAS). PedMIDAS has a maximum score of 80 (1-month version),^19^ with scores of 3 or less indicating no disability, 3–9 indicating mild disability, 10–16 indicating moderate disability, and > 16 indicating severe disability. PROMIS Sleep Disturbance questionnaire was also filled out to capture the perception of sleep quality and the PROMIS Sleep-Related Impairment questionnaire, which captured symptoms of insufficient sleep (e.g. daytime sleepiness). PROMIS rates no, mild, moderate, and severe symptoms based on T-scores.^20^ Data were also collected on chronotype and weekday versus weekend sleep/wake habits using the standardized chronotype questionnaire.^21^

Daily migraine symptoms, acute medication use, and function were recorded with a validated text-based Daily Headache Diary^22^ with additional questions to capture device wear compliance, and light-blocking lens use. Daily headache diaries were filled out via text message using the HIPAA-compliant Twilio platform hosted at CHOP. Specific questions included “Have you had a headache today” (yes or no), “Has your headache gotten in the way of your school, home, or social life today” (yes or no), and “Rate your light sensitivity (0–5).”

### Light Logger Data

#### Device wear and data collection

**Figure 1:**
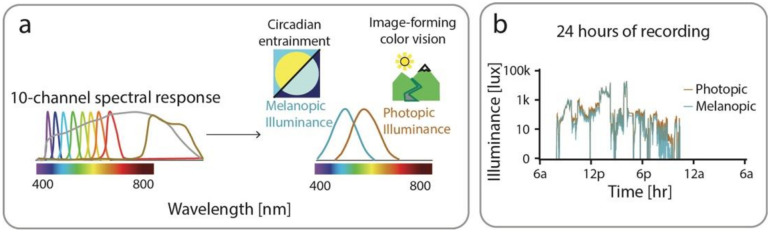
ActLumus data collection. Illustration of the ActLumus light sensor consisting of 10 Channels with different spectral sensitivities that sample at a rate of 1/minute. These measurements are differentially combined to derive melanopic illuminance that is important in circadian rhythm signaling and photopic illuminance that is the basis for image forming daylight color vision. (b) An example participant’s continuously recorded photopic and melanopic illuminance over a 24-hour recording period. Light exposure increases to measurable levels (≥ 0.01 lux) starting at 7am and then drops below measurable levels at 11pm that follows a typical 24-hour sleep/wake cycle.

ActLumus devices (Condor Instruments, São Paolo, Brazil) are wearable light loggers that provide 24-hour continuous collection of photopic illuminance and mEDI (Fig. 1). ActLumus devices were worn as a pendant around the neck, providing more ecologically valid measurements that are not obscured by shirt sleeves and are closer to the visual plane, which is critical for capturing non-image forming effects of light.^12,23^ Data were collected at a sampling rate of 1/minute to maximize temporal resolution while still providing for 7 days of continuous recording.^13^

#### Data processing

Data were visually inspected and excluded if participants reported not wearing and being away from the Actlumus device for more than 2 hours in a day. ActLumus data were pre-processed using ActiLab Software (Condor Instruments, Sao Paolo, Brazil). Specifically, melanopic and photopic lux values were derived from 10 light sensing channels that detect different wavelengths of light for every minute of data capture. The intention was to adjust measurements based on light filtering glasses wear as determined by change in light transmittance levels, but these data were not accurately measured during data collection so this step could not be pursued. Clinical data, as well as melanopic and photopic illuminance was processed using custom software developed in Matlab (Mathworks). Log transformation was performed on melanopic and photopic illuminance values to accommodate large shifts in illuminance levels.^11^

Light metrics: Two features of the visual diet were considered: 1) the intensity of light exposure, 2) the timing of light exposure. Light metrics were chosen that summarize continuous light exposure data to capture these two features of the visual diet.^11,24^ Light intensity was represented by photopic luminous exposure and time spent exposed to bright light levels. Photopic luminous exposure captures the total 24-hour photopic light exposure (kilolux*hr), which provides a measurement of overall light exposure. Bright light was defined as photopic illuminance > 1,000 lux because outdoor light ranges from 1,000 lux on a cloudy day to 100,000 lux on a bright sunny day, while indoor light is generally below 500 lux.^24-26^ Light timing was defined by percent time spent within recommended mEDI limits. A minimum 250 melanopic lux is recommended during daytime hours, while a maximum of 10 lux in the evening 3 starting three hours before bedtime, and 1 lux or less at night is recommended to support optimal timed melatonin release.^27^ These recommendations were based on expert-scientific consensus and supported by the sensitivity of human “non-visual” responses to ocular light. In this study, we chose fixed definitions of “day” defined as 7 am – 5 pm, “pre-bedtime” defined as 8 pm – 11 pm, and “night” defined as 12 am – 6 am, based on the diurnal motion of the sun and the structured schedule imposed by school. Gaps in time were left between “day,” “pre-bed,” and “night” definitions to allow for some variability across individual schedules.

#### Validation of Light Logger measurements

We validated the tabular spectral sensitivity functions of the ActLumus device. To do so, we measured a standard light source using both the ActLumus and a calibrated spectrophotometer (SpectraScan^®^ PR-670, JADAK, North Syracuse, NY). The light source was the output of an 8-channel, digital light synthesizer (CombiLED, Prizmatix, Tel Aviv, Israel) delivered via liquid light guide into a light integrating sphere (LabSphere, North Sutton, NH). The spectral radiance of the light source was measured at 2 nm resolution using the PR-670. Combining this spectrum with the ActLumus tabular sensitivity functions (provided by the manufacturer), and converting from radiance to irradiance, provided a model prediction of the ActLumus sensor counts. We then measured the light source using the ActLumus, and compared the obtained and predicted sensor counts. We found the ActLumus counts to be in excellent agreement with the prediction (Pearson’s R = 0.9982). We were, however, limited to validating 9 of the 10 ActLumus channels, as the PR-670 does not measure the infra-red sampling range of the 10th channel.

### Data Analysis

No *a priori* sample size calculations were performed as this was a pilot study to determine sample size for future studies. Descriptive statistics for continuous variables included median with interquartile range for non-normal continuous distributions and mean with standard deviation for continuous variables with normal distribution. Proportions were reported for categorical variables. Continuous light logger data was graphically represented as the mean with 95% confidence intervals (CI) determined by bootstrap analysis. Shifts in the temporal profile between weekdays and weekends and between youth with and without chronic migraine were estimated by determining the correlation between a shifted version (+/−100 minutes) of the first group and the second group and taking the highest correlation value (which was r > 0.98 for both comparisons).

## Results

### Clinical and Headache Diary data

Twenty youth with migraine participated in this study. Demographics and clinical characteristics were reported (Table 1). Participants were a median age of 17 years old [IQR 16, 19] and were 70% female, and reported a median of 17 [IQR 6, 30] days per month of any headache, and 5 [IQR 2, 15] days per month of bad headache for the previous month at the start of recording. Headache-related disability (measured by 1-month PedMIDAS scores) was moderate on average with a median PedMIDAS score of 16 [IQR 8, 29].^19,28^ Median fear-of-pain (FOPQ-C) score was 39 [31, 54], placing most youth in the moderate-to-severe range, consistent with chronic pain conditions, including migraine.^29^ All participants were on at least one, and many were on a combination of pharmacologic agents for headache prevention. Supplements, OnabotulinumtoxinA, antidepressants, and calcitonin gene-related peptide blocking agents were the most common. This is representative of patients seen in the CHOP headache clinic, who have more severe and refractory migraine compared to the general population of adolescents with migraine.

All participants had 100% compliance with headache diary prompts. Headache diary responses were consistent with validated questionnaire responses. Youth reported a median of 6 headache days [IQR 3, 7], and 3 migraine days [IQR 1, 6], indicating they had high any headache and bad headache frequency during recording week. Median daily light sensitivity score was 2 [IQR 1, 3] indicating mild light sensitivity, and median pain score was 4 [0, 6] indicating moderate pain.

### Light logging

Overall, compliance with light logger wear was high. Each participant completed 7 days of recording for a total of 140 days. For one participant, 4 days of recording were excluded due to being away from their ActLumus device for more than 2 hours and/or visual inspection was indicative of non-device wear. This left 136/140 (97.1%) of days with usable light logging data across 20 participants. Seven participants reported using light blocking lenses (blue light filtered and/or sunglasses). Of those, only one used light filtering glasses continuously, with the remainder using glasses a maximum of 1–2 hours a day on an as needed basis. The lens transmittance measurements did not record during data collection, so light exposure levels were not able to be adjusted for based on light blocking lens use.

#### 24-hour light exposure profiles

**Figure 2:**
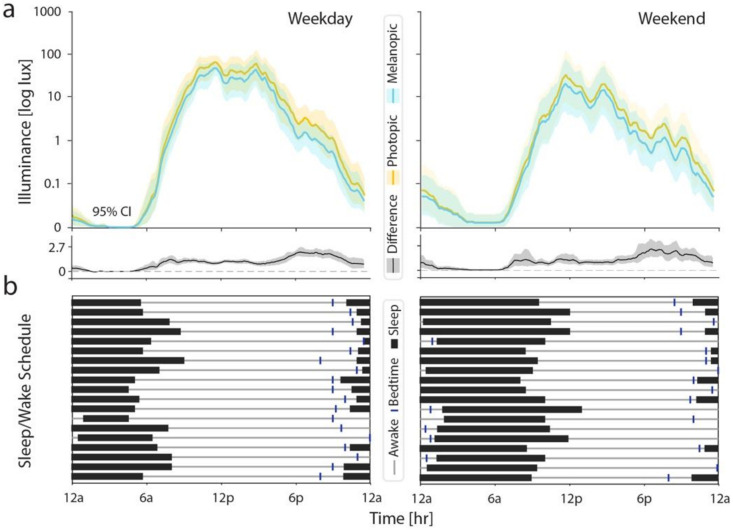
24-hr circadian light exposure patterns and sleep/wake times. (a) Photopic (orange) and melanopic (light blue) illuminance were compared for the weekdays (left) and weekends (right). The difference (photopic – melanopic illuminance) is also shown (gray). Each 1-minute timepoint was calculated across a sliding 30-minute window. The central tendency represents the mean, and error bars represent 95% confidence intervals by bootstrap analysis. (b) Reported bedtime (dark blue vertical line), sleep period (black bar), and wake times (thin gray line) reported in the Chronotype questionnaire. Each row represents one participant during the weekday (left) and weekend (right).

We evaluated patterns of photopic and melanopic illuminance across the 24-hour circadian cycle (Figure 2). We compared light exposure on weekdays and weekends, as substantial differences have been noted in adolescent populations given the structure imposed by school.^30^ Photopic and melanopic illuminance demonstrated high correlation throughout the day (Figure 2a). However, there was a relative increase in photopic compared to melanopic illuminance that was most pronounced starting around 6 pm until 10 pm. As expected, average light exposure levels were delayed by 49 minutes on the weekends compared to the weekdays. This shift was consistent with later and more variable bedtimes, sleep times, and wake-up times, and longer sleep durations reported in the Chronotype Questionnaire (Figure 2b).

### Light exposure summary metrics

**Figure 3:**
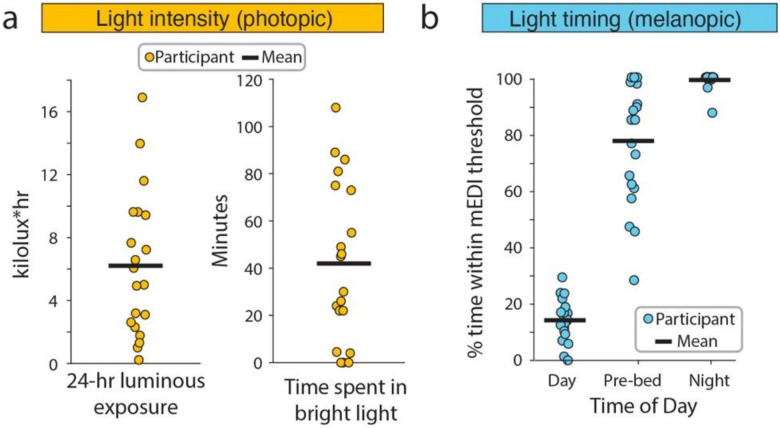
Light exposure summary metrics. (a) Light intensity. Photopic illuminance was used to calculate light intensity metrics as these have been used as the standard for designing lighting spaces. Each participant represents the mean photopic luminous exposure (left panel) and the mean time spent in bright light levels (right panel) across the 7-day period for each participant. The mean (black line) is also shown. (b) Melanopic illuminance (light blue) was used to measure the timing of light exposure because this is important for circadian entrainment. Percent time spent within recommended mEDI based on time of day: above 250 lux mEDI during the day (left), below 10 lux starting 3 hours prior to bed (middle), and below 1 lux at night during sleep (right). Mean across participants (black) and individual participants averaged across 7 days (light blue circle) are shown. mEDI = melanopic equivalent daylight illuminance.

Summary metrics were calculated to capture daily light intensity and light timing based on our findings across the 24-hr light profile, and prior studies.^11,13,16,27^ Two metrics of photopic illuminance were used to characterize daily light intensity (Fig. 3a). Total photopic luminous exposure was calculated, which represents the integrated exposure to photopic light in a 24-hour period. The mean total luminous exposure across participants was 6.2 klux*hr, with a wide range between 0.2 and 16.9 klux*hr. Time youth spent in bright light was also estimated, which was defined as light exposure of 1,000 lux photopic illuminance or greater, as indoor light is typically below 500 lux. The mean total time spent in bright light across participants was 42 minutes per day [range 0 to 108 minutes].

Percent time spent within recommended light levels across a 24-hour period was used to capture light timing (Fig. 3b). Different levels of light exposure have been recommended for healthy adults based on the time of day: a minimum light exposure of 250 melanopic lux during daytime hours; a maximum of 10 lux starting three hours before bedtime; and 1 lux or less at night is recommended.^27^ These criteria were used because similar recommendations have not been developed for the adolescent population. We therefore calculated the proportion of time within these recommended limits during daytime (7a – 5p), pre-bedtime (8a – 11p), and nighttime (12a – 6a) hours. Timing was selected based on typical school schedules, the diurnal pattern of the sun at the location of the study. These definitions were supported by the timing of light exposure observed across participants within a 24-hour period. Participants spent an average of 14.5% +/− SD 7.0% of daytime exposed to the recommended minimum mEDI of 250 lux (Fig. 3b). Percent time spent within recommended levels improved substantially in the pre-bedtime and night hours, with youth spending an average of 77.5% +/− SD 21.6% of the time the maximum recommended mEDI of light pre-bedtime, and 99.1% +/− SD 2.9% of the time during night hours.

### Chronic migraine

**Figure 4:**
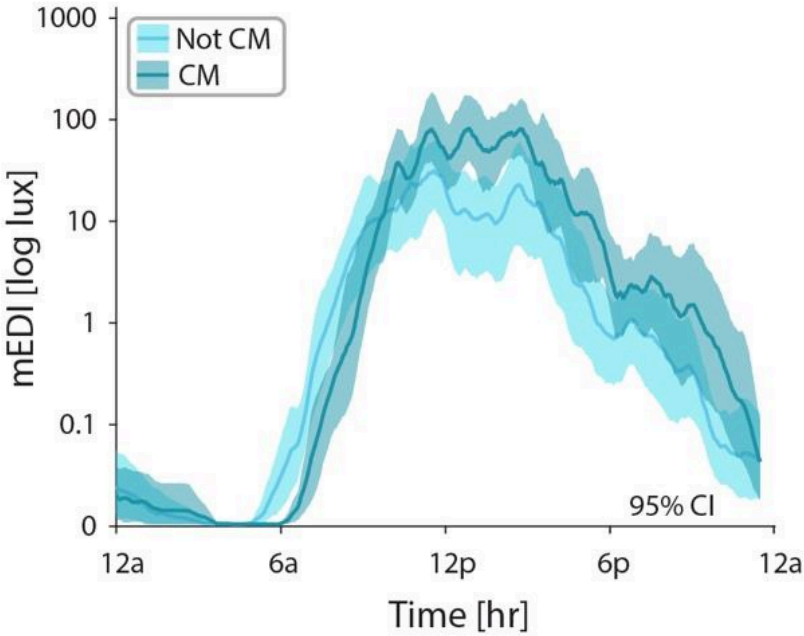
Comparison of mEDI across participants with chronic migraine defined as at least 15 headahe days per month, with at least 8 of those days being bad headache (dark teal) compared to those who did not meet criteria for chronic migraine (light teal). mEDI for each minute was averaged using a sliding window (width 30 minutes). Central tendency represents the mean. Error bars represent 95% CI by bootstrap analysis. CM = chronic migraine; mEDI = melanopic equivalent daylight illuminance.

To determine if there were any emerging differences in youth with migraine based on headache frequency, we compared the temporal profiles of melanopic illuminance of youth with chronic migraine (15 or more headache days per month with 8 or more bad headache days per month;^31^ *n* = 8) to youth who did not meet these criteria (*n* = 12; Fig. 4). Youth with chronic migraine demonstrated an average temporal profile that was delayed by 60 minutes, and a subtle increase in the intensity of melanopic illuminance compared to those without chronic migraine. A similar pattern was observed for photopic illuminance.

We did not pursue additional statistical testing as the goal of this study was to determine sample sizes needed to appropriately power future research. Instead, we conducted power analyses to determine sample sizes needed for group comparisons of youth with migraine (e.g. youth chronic migraine versus lower frequency migraine, those with high versus low headache-related disability, or high versus low light sensitivity) across light logger metrics (see *Supplemental Materials*). We found that sample sizes of 50 to 150 would be sufficient for most comparisons.

### Participant Feedback

Seventeen (85%) participants agreed or strongly agreed with the statement “I would recommend somebody to participate in this study,” while 3 (15%) strongly disagreed. Specific comments included liking the text reminders for the diary and to remember to wear the devices (1). Participants offered ways of improving the study including making the device smaller and addressing challenges with the headache diary only being once a day but experiencing multiple headache spikes a day.

## Discussion

We conducted a pilot study monitoring light exposure during everyday life of 20 youth with migraine, most with high migraine disease burden. To our knowledge, this is the first study demonstrating light exposure habits in a population with migraine using wearable light logger technology. Here, we review intriguing trends we observed in youth with migraine in the context of other studies, and how these findings should inform future study design. We found that participants spent only about 15% of the daytime (~ 1.5 hours per day) at or above the recommended minimum daylight levels for healthy adults. By comparison, they spent most of their time below the recommended maximum light levels 3 hours before bed, and during the night (78% and 99% on average, respectively). It is important to note that these recommended light levels are based on optimal light intensity and timing needed for appropriate circadian fluctuations of melanopsin. ^27^ Large observational studies using this metric are needed to determine if there is a broader impact on general health if these recommendations are not met, or are only met some of the time.

We suspect the low light exposure levels we observed in this study are due to multiple factors. Perhaps one of the largest contributors to low daytime light exposure—not unique to individuals with photophobia—is the tendency in the modern societies to spend most of the time in indoor lighting environments that are darker than outdoor environments. Lucas and colleagues conducted a similar study in 59 generally healthy, mostly younger adults, providing an important reference point. They found participants spent 33% of daylight hours at or above recommended light levels, but 66% of the time at or below recommended light levels 3 hours before bed.^16^ Results across the two studies are similar, however participants in our study were exposed to lower light levels at each point in the 24-hour cycle. Additionally, youth with migraine spent less time exposed to bright light: the adults spent an average of 1.7 hours per day under bright light conditions^16^ compared to an average of 40 minutes per day in our study. These differences are present even though Lucas and colleagues used wrist watches that can underestimate light exposure compared to pendant wear.

While it is possible that low light exposure reflects light avoidance driven by migraine-related photophobia in our study population, there are other explanations. First, it is currently unknown if adolescents behave similarly to adults in light exposure habits and normative data in younger populations are needed. Additionally, Lucas and colleagues completed recording February through July while we recorded November through March, biasing our study towards the winter months when light exposure tends to be lower. Illustrating this, a study including 15 adults in Amsterdam found a 4-fold increase in the time spent above 250 lux mEDI between the winter and summer.^11^ We also found that bright light exposure was variable across participants, ranging from 0 to 2 hours a day. This may reflect differences in other factors (e.g. participation in outdoor sports) that could influence light exposure. Clearly, simultaneous measurements of study and control populations will be needed to support stronger claims of altered visual diet in migraine.

We observed light exposure profiles shifted later on the weekends compared to the weekdays. This corresponded with reported sleep/wake times and is consistent with prior studies in adolescents.^30^ We also observed that the entire temporal light profile was shifted an hour later for youth with higher frequency migraine attacks (i.e. chronic migraine) in this preliminary sample. Later chronotype has been associated with more frequent headache in youth,^32^ which is consistent with these findings. The causal direction of these emerging associations is unknown. It is possible, for example, that youth with chronic migraine rise later due to severe symptoms, producing a shifted sleep schedule, resulting in greater artificial light exposure in the evening and before bed. These effects may also reflect sleep disruption influenced by exposure to evening and nighttime screens. This is consistent with the finding that increased headache frequency in youth is associated with prolonged screen use.^32,33^ Further study is needed to confirm and understand these associations.

### Strengths and Limitations

To our knowledge, this is the first study to report objective measurements of light exposure combined with a daily diary to track migraine symptoms in real time. We used a light logger with 10 channels of differing spectral sensitivities allowing for the separation of photopic and melanopic illuminance, while light loggers with a single channel provide only a non-specific measure of overall illuminance. The multichannel measurements allow for more mechanistic hypothesis testing. We observed that the relative strengths of photopic and melanopic illuminance diverged during the evening hours. This may reflect differences artificial lighting environments where these signals can dissociate,^34^ and highlights the importance of collecting both measurements. Overall, diary and device compliance were high providing a complete dataset for analysis, and participants generally had positive feedback on the study design.

Limitations include small sample size and the lack of a control group. Furthermore, participants were established patients of a pediatric headache clinic receiving active treatment, limiting the generalizability of the results. There may be bias in that youth willing to participate in the study may be more likely to already be working on healthy headache habits, while those still struggling with daily habits may be less likely to participate. While light filtering lens use was captured by survey data, we did not correct our measurements for the continuous use of blue light filtering glasses for one participant. Finally, data collection occurred in the winter months when light exposure is lower in the Philadelphia area, thus it is unclear if results are generalizable to different times of year.

### Future Directions

Studies that include larger sample sizes and control comparison to migraine-free peers are needed. Prior reports have offered that significant differences between winter and summer light exposure should be found with sample sizes as small as 3 individuals.^11^ Our calculations in youth with migraine indicate differences within disease states may be more subtle; we found sample sizes of 50 to 150 will be needed to detect group differences based on subjective report of migraine burden and sleep for most light metrics (see *Supplemental Materials*). Controlling for time of year is also critical as there are dramatic differences in light exposure between the summer and winter months at greater latitudes.^35^ This cycle may contribute to the seasonal variation observed in migraine, where migraine symptoms to be worse in the late fall and winter months,^36-39^ and seasonal comparison offers a unique opportunity for within-participant measurement.

Ultimately, insufficient and/or poorly timed light exposure may offer a modifiable risk factor for increased disease burden in youth with migraine. If larger observational studies confirm a relationship between migraine disease burden and light exposure, then clinical trials focused on interventions that address visual diet could be pursued. Multiple participants expressed interest in having access to their data, suggesting this may be a viable target for behavioral intervention.

## Conclusion

Like prior studies in a general adult population, youth with migraine receive less than recommended daytime light exposure but do tend to achieve recommended levels of darkness during the evening and night. Measuring daily light exposure in youth with migraine is feasible and promising avenue for larger observational studies.

## Figures and Tables

**Table 1:** Participant demographics, baseline headache characteristics, and treatment.^a^For the two participants who indicated they were Hispanic, both selected “prefer not to answer” for race. ^b^Sleep impairment measures symptoms of poor sleep including fatigue and daytime sleepiness, while sleep disturbance measures difficulties falling and staying asleep. PedMIDAS = Pediatric Migraine Disability Assessment, moderate/severe was defined as a score of 10 or greater. FOPQ-C = Fear of Pain Questionnaire for Children, moderate/severe is defined as a score of 30 or greater, based on FOPQ-C definition; VLSQ-8 = visual light sensitivity questionnaire, moderate/severe light sensitivity was defined as a score > 24, which is the midpoint score.

Demographics and Headache Characteristics			
Age [IQR]		17 [16, 19]	
Sex *n* (%)		F 14 (70), M 6 (30)	
Race Ethnicity	Hispanic^a^ *n*(%)	2 (10)	
	Non-Hispanic Black *n* (%)	2 (10)	
	Non-Hispanic White *n* (%)	15 (75)	
	Pref. not to answer *n* (%)	1 (5)	
Validated questionnaires	Headache d/mo. [IQR]	17 [6, 30]	
	Bad Headache days/month [IQR]	5 [2, 15]	
	Continuous headache *n* (%)	12 (60)	
	PedMIDAS (1 mo.) [IQR]	16 [8, 29]	
	Moderate/Severe *n* (%)	14 (70)	
	FOPQ-C [IQR]	39 [31, 54]	
	Moderate/Severe *n* (%)	16 (80)	
	VLSQ-8 [IQR]	23 [19, 27]	
	Moderate/Severe *n* (%)	7 (35)	
	PROMIS - Sleep	Impairment^b^	Disturbance^b^
	None (%)	7 (35)	10 (50)
	Mild *n* (%)	3 (15)	1 (5)
	Moderate *n* (%)	4 (20)	4 (20)
	Severe *n* (%)	6 (30)	5 (25)

## Abbreviations

CHOP: Children’s Hospital of Philadelphia
ICHD-3: International Classification of Headache Disorders 3^rd^ Edition
IQR: interquartile range
mEDI: Melanopic Equivalent Daylight Illuminance
SD: standard deviation
